# Repetitive regime of highly focused liquid microjets for needle-free injection

**DOI:** 10.1038/s41598-020-61924-0

**Published:** 2020-03-19

**Authors:** Jan Krizek, Paul Delrot, Christophe Moser

**Affiliations:** School of Engineering, Swiss Federal Institute of Technology in Lausanne (EPFL), Station 17, 1015 Lausanne, Switzerland

**Keywords:** Drug delivery, Applied optics, Biomedical engineering

## Abstract

Fast liquid jets are investigated for use as a needle-free drug delivery system into an elastic tissue such as skin. Using smaller jet diameters in a repetitive regime can mitigate bruising and pain associated with current injectors. In this study, we aim to unravel the potential of the method to deliver liquids into biological tissues having higher elasticity than healthy skin (i.e >60 kPa). To address this challenge, we have implemented a laser-based jetting system capable of generating supersonic liquid microjets in a repetitive regime. We provide insights on the penetration of microjets into hydrogel samples with elastic modulus ranging from 16 kPa to 0.5 MPa. The unprecedented speeds of injection (>680 m/s) together with a newly introduced repetitive regime opens possibilities for usage in needle-free drug administration into materials with elasticity covering the wide spectrum of biological soft tissues like blood vessels, all skin layers, scarred or dried skin or tumors.

## Introduction

Development of needle-free delivery systems for distribution of medicines was listed as one of the grand challenges in global health^1^. The goal is to reduce contaminated waste, mitigate the risk of disease spreading or to avoid needle-stick accidents. Fast liquid jets carry spatially confined kinetic energy that can penetrate into a tissue^2^. Motivation for this work ranges from the development of needle-free transdermal drug injectors^3–9^, “liquid scalpel” for soft tissue dissecting^10–12^ or devices for gene delivery^13–15^. The main advantage lies in the smaller extent of collateral damage caused to the tissue, superior lateral precision and addressability of certain depths^16^.

The use of currently available jet injectors is associated with pain and bruising which originates from the relatively large injection depth and volume^4^. This can be addressed by injecting smaller diameter jets in repetitive regime thus compensating for the smaller volume of each jet by its multiplicity. In the context of needle-free drug delivery, this strategy was first implemented by Arora^4^ using a piezo actuator system^17^. The Arora study^4^ showed that *in vivo* injection in rats minimized the collateral damage and provided sufficient dosage of insulin. The same technology was used by Römgens *et al*.^3^ who reported a delivery efficiency through the first skin layer (epidermis) as high as 90%. Jets were mostly stopped by the reticular dermis and reached the hypodermis with the efficiency of only 12%. With regard to the diagram presented by Rodríguez *et al*.^18^ we can claim, that such shallow penetration prevents this technology from delivering a drug aimed to reach the hypodermis or muscle tissue.

The application of microjets in neurosurgery is driven by the absence of thermal damage and preservation of small blood vessels^19^. Extensive work in this field has been done in the group of Prof. Takayama^12,19^. Their device uses laser pulses coupled to an optical fiber which is inserted in a liquid filled nozzle. A laser-induced cavitation bubble pushes out the liquid in the form of a jet. This laser induced liquid jet system has proven to be a useful tool for neurosurgical applications in several clinical studies^12^. Fletcher and Palanker^10^ generated pulsed liquid microjets driven by an electric discharge-induced vapor bubble inside a micronozzle and demonstrated significantly smaller incision diameter by liquid jets compared to the direct application of a plasma-induced vapor bubble.

Schramm-Baxter and Mitragotri^20^ showed that the penetration depth in hydrogel samples can be described by the jet power, which yields a square dependence on the jet diameter *D* and a cubic dependence on the jet velocity ${v}_{jet}$*:* $P\propto {D}^{2}{v}_{jet}^{3}$. Therefore, the jet velocity has a bigger impact on a penetration depth than the diameter. Typical jet velocities of needleless injectors based on pressurized gas, string actuation or piezo crystal actuation are up to 150 m/s. In addition, these conventional needleless devices generate micro-jets with a diffusive shape^2^. On the other hand, highly-focused supersonic micro-jets (>850 m/s) have recently been demonstrated by Tagawa *et al*.^21^ using a laser-induced flow focusing phenomenon. In this system, a nanosecond laser pulse induces a shockwave that impacts a concave meniscus, thus creating a flow-focused micro-jet tip whose shape remains spatially focused even for high Reynolds number (the highest measured Reynolds number is Re ≈ 10^4^ by considering the effective length-scale as the diameter of the tip which is 0.1 of the capillary diameter). Moreover, the thin shape has a positive influence on the penetration performance and lateral positioning of the dose^16,22^.

Here, we demonstrate supersonic flow-focused micro-jets in a repetitive regime. In this way, we combine the advantage of generating high velocity jets (>608 m/s) together with a multiplicity of jets that enables penetration into harder samples while providing a proper dosage and controlled penetration depth.

We carried out experiments by measuring the injection depth dependence on jet speed (<1072 m/s), jet diameter on the tip (10, 15, 30 µm). Previous works focused on the penetration into soft sample gels mimicking tissues with elastic modulus up to 10 kPa (5–10% Gelatin). We were able to map the required jet power for different elasticity and the results suggest potential usage of this technology for needle-free injection into materials with elastic modulus ranging 13–450 kPa, covering the wide spectrum of biological elastomers like inner body organ tissues, blood vessels, all skin layers, or tumors^23–27^.

## Materials and Methods

### Generation of fast microjets

**Figure 1:**
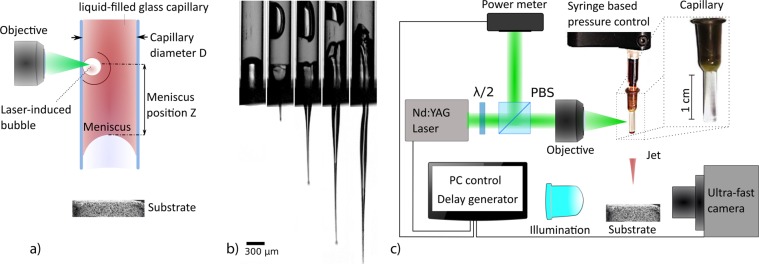
(**a**) Laser actuation scheme. A laser-induced bubble pushes the liquid out of the nozzle that is further accelerated thanks to the flow focusing effect occurring at the liquid/air meniscus shaped interface. (**b**) Liquid jet snapshot sequence from the ultrafast camera (60000 fps). Flow focusing causes high acceleration and the sharp geometrical shape of the liquid jet. The shape is roughly invariant with a jet speed. The diameter of the jet tip is approximately 1/10 diameter of the capillary. The droplet on the tip is observed only in the slower jets up to 100 m/s (**c**) Scheme of the set-up. The laser pulse (5 ns, 532 nm) is split by the polarization beam splitter (PBS), which allows online monitoring by the energy meter. Further, it is focused (20× objective) into a round capillary filled with a water-based ink. The generated jet, its velocity and the penetration depth into a transparent receiving substrate is monitored by an ultra-fast camera. Control of the meniscus position is performed by the custom-made syringe based pressure control.

Figure 1a illustrates the mechanism of laser-based actuation with all relevant parameters. Previous works described the dynamics of laser-induced flow focusing^21,28^. At time *t* = 0 μs, a laser pulse is fired and the subsequent absorption of energy by the liquid leads to a rapid cavitation. The evoked pressure wave propagates in the axisymmetric liquid environment until it encounters the liquid/air interface. The curvature of the meniscus, caused by the capillary-glass wetting, results in the local orientation of the pressure toward the axis which contributes to a focusing effect of the liquid flow (Fig. 1b). The diameter of the jet tip is measured to be approximately 1/10 of the capillary diameter.

### Set-up

The experimental setup is shown in Fig. 1c. Jets are actuated by a 5-ns laser pulse (Continuum, ML-II, 532 nm) focused by a 20x microscope objective on a liquid filled borosilicate glass capillary with inner diameters 300, 150 and 100 μm (CM Scientific Ltd.) for generation of the jets of 30, 15 and 10 µm respectively. The distance between the meniscus interface and laser focus *Z* was kept twice the capillary diameter, which we found as a minimum for having a jet with the same direction as the capillary axis. To prevent breakage of capillaries due to the optical pulse or generated pressure, we use a thick wall capillary with the outer diameter of 3 mm. The meniscus position in the capillary is controlled by a custom-made syringe based pump in case of single shot experiments. For jetting in repetitive regime, we use the Syringe pump (New Era Pump Systems Inc., NE-1000) with customizable flow rates. Capillaries are tightly sealed to the syringe connector without tube connectors to minimize flow rate settling time. To monitor the optical energy, we split the pulse by the polarization beam splitter (PBS) where the splitting ratio is given by the polarization angle adjusted with a half-wave plate $(\frac{\lambda }{2})$, measurement is done by a power meter (Thorlabs, ES111C). The imaging of the jet formation, jet speed and transparent material penetration is performed with an ultra-fast camera (Vision Research, Phantom Miro M310), which requires a back illumination of the scene to obtain a good contrast image (custom-made high brightness source). The minimum time between two frames in our experiments is 5 µs (200 kfps). For the velocity measurement we use the objective with the magnification 2×, 4×, 6× for measurement of *30, 15, 10* µm jets respectively. Synchronization of the laser and the camera is performed by the delay generator (Berkley Nucleonics, Model 577). To enhance the laser light absorption at the wavelength of pulsed light source (532 nm), we stained the liquid with *52* mM of Allura Red AC (ARAC; 80%, Sigma Aldrich, USA). The absorption coefficient of the solution was measured as ${\epsilon }=70\cdot {10}^{3}\,{m}^{-1}$. The standoff distance between hydrogel substrate and the initial meniscus is kept constant at *1.5* mm, which is still within the range where the jet keeps its asymptotic velocity^21^ and does not undergo significant deceleration.

### Jet speed and power

The role of relevant parameters on the jet velocity ${v}_{jet}$ was described in previous studies^21,28^. Assuming we vary only the capillary diameter ${D}_{cap}$ and the laser energy $E$, we will approximately obtain the following relation: ${v}_{jet}\propto (E-{E}_{th})/{D}_{cap}$. Where ${E}_{th}$ is the minimum optical pulse energy required for a jet generation which is itself a decreasing function of the capillary diameter^21^. Low energy regime (<100 µJ) was not explored in this work, because under certain value we did not observe a well-defined jet for all tested capillary diameters. Our own experiments and simulations by Peters *et al*.^28^ suggest that linearity is preserved until a certain low energy threshold is reached. The threshold energy value depends on thermal effects^29^ and the potential energy represented by the surface tension and inertia with respect to the generated kinetic energy. It also depends on the spatial and temporal profile of the energy absorption.

Combining the empirical expression for the jet velocity found by Tagawa^21^ with the equation for the power of the jet derived by Schramm-Baxter and Mitragotri^20^, we obtain the following expression for the jet power (see the Appendix 0):

1 $${P}_{jet}=\frac{1}{8}\rho \pi {D}_{jet}^{2}{v}_{jet}^{3}\propto \frac{{(E-{E}_{th})}^{3}}{{D}_{cap}}$$

From this relationship, we see that by using the same optical pulse energy *E*, we can achieve higher jet powers ${P}_{jet}$ simply by using a smaller diameter capillary.

**Figure 2:**
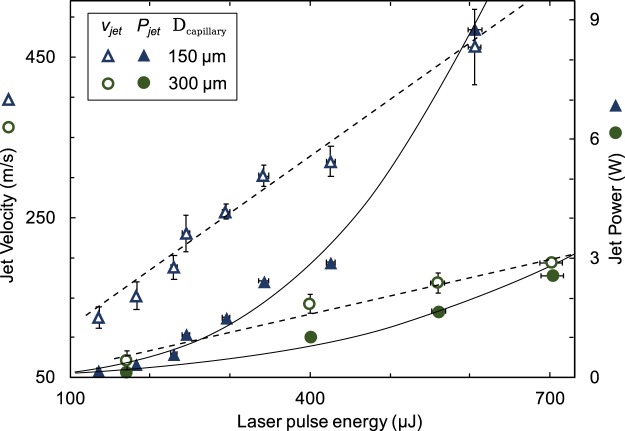
Time-of-flight measurement of the jet velocity from the ultrafast camera for two capillary diameters (150 µm and 300 µm). Jet velocity is a linear function of the pulse energy (Linear fit: *v*_150_ = *0.71*^.^*E* + *36*, *v*_300_ = *0.23*^.^*E* + *40*). Jet power follows a cubic relation to laser energy (Cubic fit: *P*_150_ ≈ 3^.^10^*−8*.^*E*^3^, *P*_300_ ≈ 4^.^10^*−9*.^*E*^3^). Error bars represent standard deviation from at least 5 velocity measurements of different jets initiated by the same laser energy.

Experimental results in Fig. 2: Time-of-flight measurement of the jet velocity from the ultrafast camera for two capillary diameters (150 µm and 300 µm). Jet velocity is a linear function of the pulse energy (Linear fit: *v*_150_ = *0.71*^.^*E* + 36, *v*_300_ = *0.23*^.^*E* + 40). Jet power follows a cubic relation to laser energy (Cubic fit: *P*_150_ ≈ 3^.^10^*−8*.^*E*^3^, *P*_300_ ≈ 4^.^10^*−9*.^*E*^3^). Error bars represent standard deviation from at least 5 velocity measurements of different jets initiated by the same laser energy. It confirms that the jet velocity is a linear function of the pulse energy (Linear fit: ${v}_{150}=0.71E+36$, ${v}_{300}=0.23E+40$). We applied the Eq. (1) and calculated the jet power to illustrate different jet power increase with respect to the laser energy in case of two different capillary diameters.

The highest velocity we were able to measure with our camera was 680 m/s. Higher jet velocities are too fast to be caught in at least two subsequent camera frames. Theory and experimental observation suggest^1^, that the linear dependence remains at least until the speed approaches the speed of sound in water ≈ 1500 m/s). And therefore higher velocity values can be extrapolated from the linear fit obtained with a slower jet measurement. Linearity is also not preserved at the low energy where surface energy and inertia dominates over generated kinetic energy from expanding bubble. Curvature of the meniscus has an impact on the jet velocity^21^, contact angle between borosilicate glass and the dyed solution was measured to be 30° ± 3° and kept same during experiments.

### Operational condition for the repetitive regime

**Figure 3:**
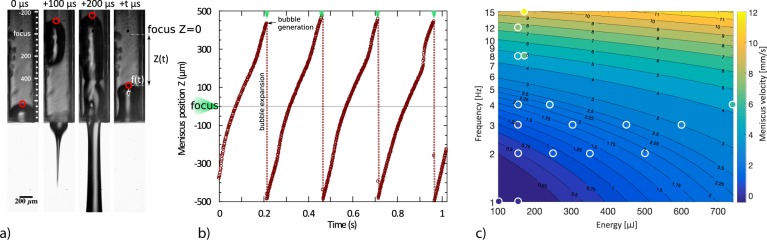
(**a**) Rapid bubble expansion and liquid ejection leaves the system in an imbalanced state after the cavitation and the jet creation. (**b**) The meniscus position tracking in the repetitive regime. The time required to restore the meniscus into its initial position is the minimum delay necessary between two subsequent jets (**c**) Operational condition for stable jetting. Color of data points reveals their actual value. Isocurves shows the value of required meniscus velocity and are based on the plane fit of data points: $\dot{Z}(t)=0.0042E+0.71f-1.554$.

A stable jetting regime is obtained when the flow-focused jet (see Section 2.1) is periodically repeated. Rapid bubble expansion and the liquid ejection leave the system in an imbalanced state after each pulse event. This can be seen in Fig. 3a. Generated bubble expands into two principal directions; on one side the liquid is pushed from the nozzle in the form of focused jet (100 µs Fig. 3a); on the other side there is a newly established interface which is pushed back into the capillary (marked by the red dot in the 200 µs Fig. 3a). We tracked the meniscus position after each jetting event. An example of the meniscus position versus time is shown on Fig. 3b. The time required to restore the meniscus into its initial position is the minimum delay necessary between two subsequent jets, that is to say between two laser pulses. The flow-focusing effect and the high acceleration occur only in the case of a fully recovered meniscus interface at the time of the next pulse. The dynamics of the jet ejection strongly rely on the distance between the optical focus and the actual position of the meniscus *Z(t)*. Therefore, careful adjustment of the inflow rate has to be set to establish stable repetitive jetting. In Fig. 3c, we show the operational condition parameter space for laser energy *E* and laser pulse frequency *f*. Experimental data points show the syringe pump setting necessary for stable repetitive jetting for given laser energy and frequency. Instead of flow rate, we use the meniscus advancement velocity $\dot{Z}(t)$ of the water in the 150 µm capillary channel in steady state. Meniscus velocity is the variable normalized over the system microfluidic properties that includes viscosity of the liquid, diameter or shape of the capillary and microfluidic resistance of the whole system. We measured experimentally that the required meniscus velocity is proportional to the jetting frequency and the laser pulse energy. We assume that these parameters are independent and by fitting obtained data we get the following expression: $\dot{Z}(t)=0.0042E+0.71f-1.554$ which describes the operational condition for stable repetitive jetting.

### Hydrogel samples

Injection experiments were made on transparent PEGDMA hydrogel samples having similar elasticity to natural skin tissue layers (see Table 1). Four hydrogel (Polyethylene glycol di-methacrylate - PEGDMA) precursors were synthesized by dissolving PEGDMA 20 kDa (5 wt%), PEGDMA mix kDa (10 wt%), PEGDMA 20 kDa (10 wt%), and PEGDMA 8 kDa (20 wt%), in phosphate buffered saline (PBS, pH 7.4) and Irgacure 2959 (0.1 g.ml-1%). The degassed mixture was then poured into specific molds made of Teflon and covered with microscope slides. The PEGDMA molecules were crosslinked by ultraviolet irradiation with a light intensity of 5 mW.cm^−2^ (365 nm) for 30 minutes. Our hydrogel samples were evaluated using the *Instron E3000* linear mechanical testing machine (Norwood, MA, USA) with a 250 N load cell and a constant speed of 0.5 mm.s^−1^. The elastic modulus is calculated in the strain range of 0–20%. The elastic modulus of the hydrogels was evaluated in unconfined compression and on the cylindrical samples with the diameter 8 mm and height of 4.5 mm. Obtained values are respectively *13.1* ± *2.6* kPa, *69.5* ± *4.8* kPa, *82.4* ± *6.9* kPa, *462.1* ± *17.2* kPa.

**Table 1:** Hydrogel samples parameters and resemblance with the body tissue.

Elastic modulus	Tissue	Hydrogel sample	Elastic modulus
1–20 kPa	Hypodermis^27^, Muscle^23^, Fat^24^	1. PEGDMA 20 kDa (5 wt%)	13.1 ± 2.6 kPa
20–100 kPa	Full thickness skin^27^, Dermis^25^	2. PEGDMA mix kDa (10 wt%)	69.5 ± 4.8kPa
		3. PEGDMA 20 kDa (10 wt%)	82.4 ± 6.9 kPa
100 kPa – 500 kPa	Viable epidermis (lower range)^25^, Stratum corneum (upper range)^25^, Dehydrated skin^25^, Cancer tissue^24^	4. PEGDMA 8 kDa (20 wt%)	462.1 ± 17.2 kPa

## Results and discussion

### Material penetration and delivery efficiency

**Figure 4:**
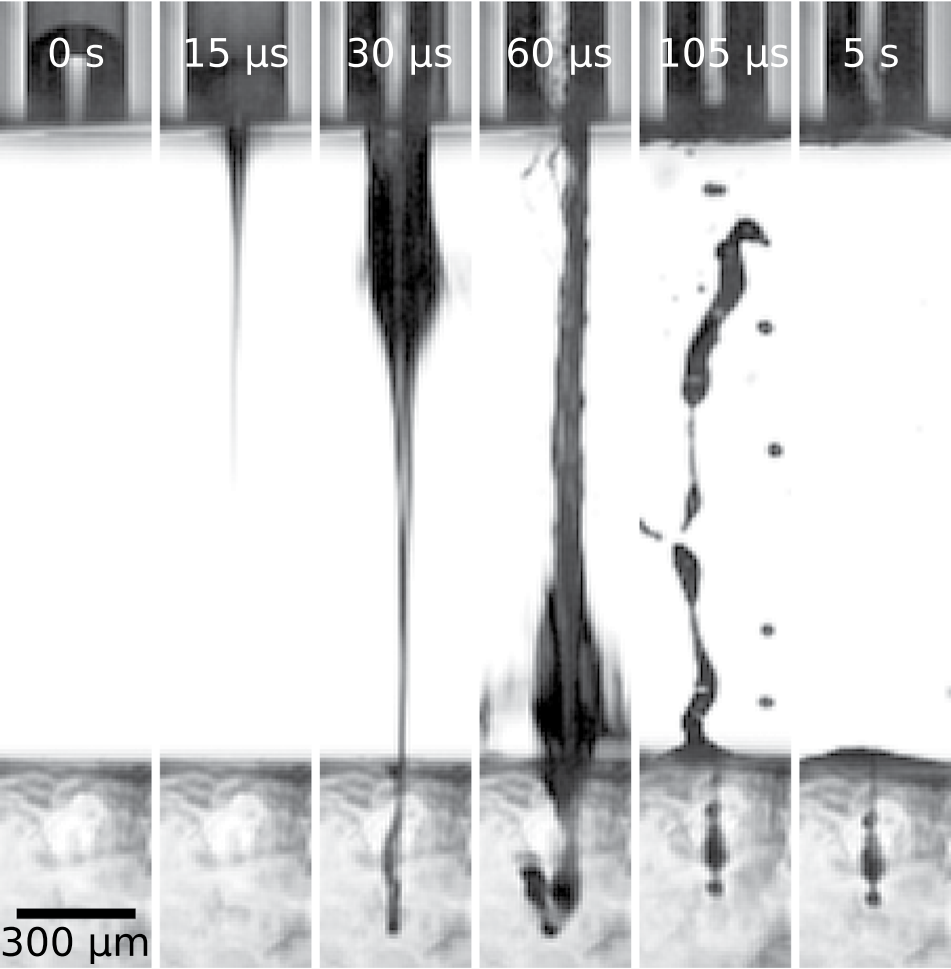
Typical injection sequence into the transparent hydrogel sample. On the picture, ${v}_{jet}=238\pm 10\,m/s$ ø_*jet*_ = 30 μ*m*, ${P}_{jet}=6.3\,W$, hydrogel elastic modulus $69.5\,\pm 4.8\,kPa$.

We can visually monitor the injection depth with the camera. A typical injection sequence is shown in Fig. 4. There are two main factors determining the success of injection: jet power and material property. Problem with assessment of the efficiency lays in uneven distribution of the velocity as the acceleration is driven by two actions – flow focusing (focused jet tip) and bubble expansion (the slower dispersed tail). This inhomogeneous velocity distribution affects the delivery efficiency since stiffer materials can be only penetrated by the fast focused part (≈10 nl for ø_jet_ = 30 µm), which stands for only few percent of the whole ejected liquid. Efficiency of the system depends greatly on whether the velocity of the slower tail exceeds a critical value for given material stiffness, the efficiency can be above 90%, as it was referred in^22^. For the same material, jets with higher power are more efficient as larger part of the jet exceeds critical velocity. The aim of this manuscript is to elucidate critical parameters for high velocities and stiff materials. That is why we elaborate only on the focused part of the ejected fluid because for the high material stiffnesses we are not able to generate slower tail part fast enough to penetrate in. System avoiding slow-tail are realized by a change in the generation scheme in a way the laser pulse is close to the interface like in Turkoz *et al*.^30^, Delrot *et al*.^16^, or Hayasaka *et al*.^31^. In this paper we elaborate only on the critical values required to achieve certain depths without the assessment of the efficiency. This information is important for controlled delivery into certain layers of skin and therefore targeting certain cell types^14^ and also revealing the potential for penetration into stiffer tissue.

### Depth of the penetration with different capillary diameters

**Figure 5:**
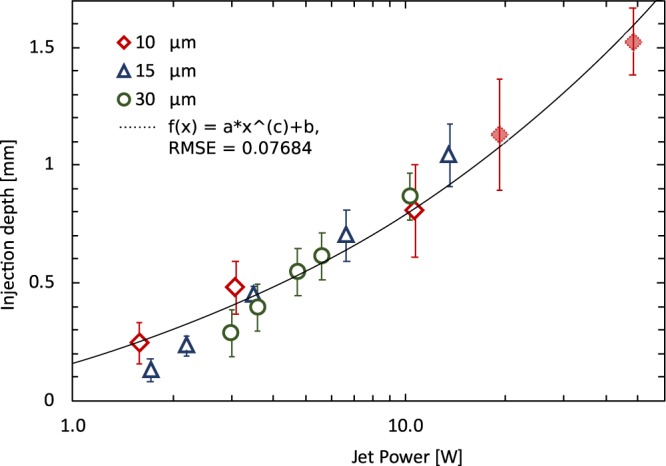
Injection depth of the single jet as a function of the jet power (power is derived from the measurement of velocity). Experiments are done with the same material (Young’s modulus 69.5 ± 4.8 kPa) for the different capillary diameters resulting in jets of diameters 10, 15, 30 µm. The injection depth increases linearly with the increasing jet speed (data not shown), therefore the depth increases with the cube root of the power (shown in the log scale). Black line represents best fit of the cube root function $Depth\approx 560\sqrt[3]{{P}_{jet}}-411$, RMSE = 0.07613. Error bars are calculated as standard deviation from at least 7 measurements. Jet velocities of data points with filled markers were extrapolated from the measurement of slower jet.

Schramm-Baxter and Mitragotri^20^ showed that jet power (Eq. (1)) is an effective predictor of penetration depth in their experimental conditions - diffusive jet shape, jet diameter 76 µm–559 µm, jet velocity 115 m/s–200 m/s. In our case, we hypothesized that this relation also holds for jets generated by laser actuation and with the flow focusing effect. This was experimentally confirmed (see Fig. 5) by measuring the relationship between injection depth and the jet power for focused jets with diameters 10–30 µm and with velocity ranging from 200 m/s to 1072 m/s. The power is calculated using the Eq. (1) and counting with the jet velocity measured by time of flight. We note that the assumption of jet power being the single determining factor is not trivial since the penetration mechanics of focused jets^22^ is different from that of diffusive jet shapes referred in Schramm-Baxter and Mitragotri^20^. Our experimental measurements for injection depth nonetheless show a linearly increase with jet speed, therefore the depth increases with the cubic root of jet power. This result supports the hypothesis of Section 2.3 which states that for a given laser energy, smaller capillaries yield greater penetration depth.

The use of jets with diameters smaller than 100 µm is the current trend in the field because of decreased collateral damage as mentioned before^2,3^. In the context of our set-up, a smaller capillary diameter reduces the required laser energy. This experimental data also suggests that the injection depth can be controlled by tuning the laser actuation energy.

### Injection depth for different material elasticity

**Figure 6:**
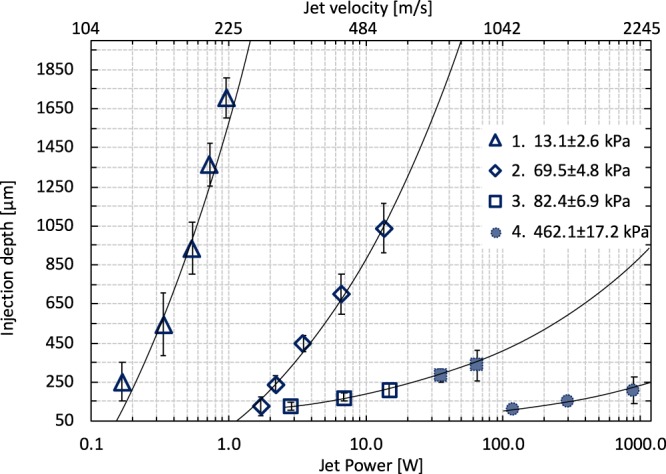
Injection depth of the single jet into the hydrogel samples of different elasticity matching the range of soft tissue materials − 1. – fat-like tissue, muscles, inner organs; 2.,3. – dermis, full thickness skin; 4. – epidermis. Tests were made with the same diameter of the jet ø_jet_=15 µm. Black line represents best fit of the cube root function $Depth\,1.\approx 3400\sqrt[3]{{P}_{jet}}-1725$, $Depth\,2.\approx 756\sqrt[3]{{P}_{jet}}-741$, $Depth\,3.\approx 90\sqrt[3]{{P}_{jet}}-3$, $Depth\,4.\approx 23\sqrt[3]{{P}_{jet}}-10$. Error bars are calculated as standard deviation from at least 7 measurements. Jet velocities of data points with filled markers were extrapolated from the measurement of slower jet.

All tests described below were made using a capillary with a diameter of 150 µm. Figure 6 shows the injection depth results in materials having an elastic modulus of 13 kPa, 69 kPa 82 kPa and 462 kPa. As expected, the elastic properties of the material plays a crucial role for injection characteristics, and we also observe a penetration threshold for a given elasticity^12^. The graph in Fig. 6 gives an estimate of the jet power necessary for injection into a specific tissue. The first data point of each measurement represents the power required so that 7 out of 7 jets penetrate into the hydrogel sample. Fat-like tissue in the lowermost layer of the skin or muscles (E < 20 kPa^23,27^) can be penetrated by jets with power above *0.1* W. For tissues mimicking the elasticity of dermis, which is the middle skin layer (E < 100 kPa^25,27^), a jet power of at least *1* W is required, while injecting through the full skin thickness (≈1 mm^18^) requires a jet power exceeding *100* W. The outermost layer of the skin – epidermis - is creating a physical barrier and is the stiffest skin layer (E ≈ 500 kPa^25^). We found the penetration threshold power for materials in this elasticity range, to be above 100 W and the depth increase slowly with jet power. Interestingly, we also observe a large difference between sample 2 and 3 which both have similar elasticity (69 kPa vs 82 kPa) but are synthetized with a different precursor batch (different precursor polymer chain length).

This points to the high sensitivity of penetration depth on material properties and underlines the need of adjusting the micro-jet injector to the different skin types which naturally possess different elastic modulus depending on the individual subject and conditions^32^. Furthermore, cancerous tissues are in general stiffer^23^ than healthy ones, thus we can expect that drug delivery into a skin cancer site^33^ would require higher power jets. We further show in the next section that penetration depth can be increased by implementation of a repetitive regime, but the minimal jet power must exceed the threshold for penetration. High sensitivity also underlines the need for inspection of the real tissue samples with its heterogeneous structure.

### Repetitive regime

The jet repetition rate of our system is limited by the laser source pulse frequency to *f* = *1–15* Hz. We hypothesize that by using higher-repetition laser source, it could be extended up to the frequency limit similar to an inkjet printer head in the kHz range. The highest measured jet velocity in the repetitive regime was *605* m/s (*P* = *20* W) for the *150* µm capillary. This limitation is given by the camera acquisition speed. We observed jetting with actuating pulse energy of 3 mJ for which we can expect the jetting speed to exceed the speed of sound in water (1500 m/s). Jet speed fluctuation from jet to jet was below 10%. We observed stable jetting for as long as 5 minutes (*f* = 1*5* Hz, ≈ *4500 jets*, ≈ *50* nl *– counting only focused tip of the jet)*.

Table 2 provides an overview of pulsed liquid microjet generators published in the literature. Jet generators for surgical dissection^10–12^ have single jet powers up to *1* W. For needle-free drug delivery systems, the required jet power increases due to the higher elasticity of the first skin layer^3,4^. In comparison, this work shows unprecedented jet power in the repetitive regime. Lower volume can be compensated by a high-frequency rate or by increasing the drug concentration as our jetting technique is capable of dealing with high viscosity liquids^34^.

**Table 2:** Comparison table of technologies for the generation of repetitive liquid micro jets.

	Technology	*	Velocity [m/s]	Diameter [µm]	Power [W]	Frequency	Volume
Nakagawa *et al*.^12^	Laser	S	10	500	**0.1**	10 Hz	20 nl
Fletcher and Palanker^10^	Electrical breakdown	S	90	30	**0.3**	?	100 pl
Lü *et al*.^11^	Laser	S	25	300	**0.6**	5 Hz	
Römgens *et al*.^3^	Piezo-actuator	D	130	50	**2.2**	1 Hz	20 nl
Arora *et al*.^4^	Piezo-actuator	D	127	100	**8.0**	1 Hz	10 nl
This work	Laser+Flow focusing	—	605 (2000)	15	**19.8** (700)	15 Hz	[pl]

*S – surgical purpose, D – drug delivery. Velocity value listed for our device is the highest measured velocity. We hypothesize that that this value is much higher while actuating with higher laser energies. Values in brackets represent extrapolation based on measurement of slower jets for the highest laser energy we realized consistent jetting (3 mJ).

#### Penetration depth with repetitive regime

**Figure 7:**
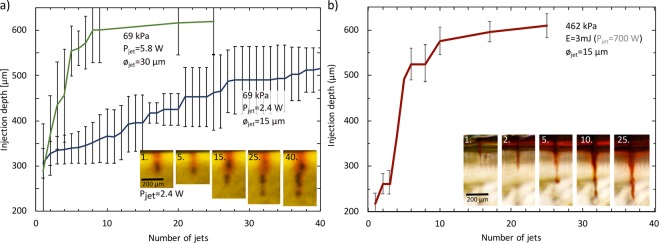
Injection depth of the jets in repetitive regime into hydrogel samples with images of injection site. (**a**) *Young’s modulus* = *69.5* kPa (**b**) *Young’s modulus* = *462* kPa. Error bars are standard deviation of four (69 kPa) and two (462 kPa) measurements. The final penetration depth is established in between 2–3 times of the single jet penetration depth.

Once the minimum threshold power for the material penetration is reached, we can increase the injection depth and volume by jetting in a repetitive regime. The first jet undergoes the same mechanics as mentioned in previous sections (4.1., 4.2., 4.3.) and according to its power, it penetrates the gelatin up to a certain depth. The following jet experiences lower mechanical barrier as it is going into an already pierced hole and therefore penetrates deeper. It is subjected to deceleration by the viscous forces from the present fluid and reaction force of the hydrogel induced by its expansion^35^. The final penetration depth is established after a certain number of jets (see Fig. 7) as the repulsive force grows with the depth. The *5.8* W power jet reaches the final depth after 10 repetitions in the hydrogel sample with Young’s modulus of *69,5* kPa. The *2.4* W power jet delivered into the same hydrogel reaches depth saturation after approximately 25 repetitions. After reaching the final depth, further jetting contributes mainly to increasing the injected volume as we can see on the comparison of injection images after the 25^th^ and the 40^th^ jet on Fig. 7a, bottom right. Thanks to the repetitive regime, a significant increase of the injection depth was achieved on the stiffest hydrogel sample (*E* = *462* kPa) that can be seen on Fig. 7b. Whereas a single 700 W jet achieved 200 μm penetration, 25 repetitions at the same power increased the penetration to 600 μm. In general, we observe that the depth can be increased by a factor 2–3 times compared to the injection depth obtained with a single jet. The injection depth saturates after a number of jets that varies with material properties and jet power. Cracking of the gel and viscous forces inside of the injection hole are stochastic which explains relatively large error bars. This is apparent especially at the initial stage (<10 jets) of the relative combination soft gel/high power and the jet diameter of 30 µm (69 kPa, P_jet_ = 5.8 W). Jet can be deflected inside the hole and create another channel, then the resulting depth will vary with each iteration. We can expect that this stochastic behavior will be even stronger for the case of real inhomogeneous biological tissue and the lack of precision would need to be accounted for.

## Conclusion

We show that the penetration depth of high-speed focused microjets can be described by a single variable: jet power. Control of the jet parameters allowed us to map the necessary jet power required for the penetration into materials having an elasticity range matching that of human body soft tissue (<0.5 MPa). Materials with elastic modulus <20 kPa, corresponding to fat-like tissue in the lowermost layer of the skin or muscles (<20 kPa) can be penetrated by jets with power above 0.1 W. For materials with elastic modulus <100 kPa mimicking the elasticity of dermis, a jet power of at least 1 *W* is required, while injecting through a stiff material having elasticity of 0.46 MPa requires a jet power of 700 W. We have shown that by adding a repetitive regime to the jet (up to 15 Hz), the injection depth could be further increased by a factor 2–3 times. With the highest repetitive jet power of our experiments (700 W), a penetration depth of 600 μm could be achieved in a material with stiffness 0.46 MPa. Our study suggests, that for tissues with higher elasticity (dry skin, scar tissue, cancerous tissue), the current generators of repetitive micro-jets are not applicable due to their low jet powers. The repetitive regime for laser-induced liquid microjets provides 2 orders of magnitude higher jet power compared with the current state of the art and opens up new opportunities to deliver liquid content into tissues with higher elastic modulus than it was previously possible.

## Appendix

### Jet power

The role of relevant parameters on the jet velocity is empirically described in Tagawa *et al*., 2012^21^ as follows:

2 $${v}_{jet}\cong {C}_{0}\frac{(E-{E}_{th})(1+\beta \,\cos \,\theta )}{ZD}$$

This relation shows that the contribution of the absorbed laser energy *E is* lowered by the threshold energy value *E*_*th*_ which itself is also an increasing function of the tube diameter *D*, the distance between the meniscus and the laser focus *Z* and the curvature of the air-liquid interface $(1+\beta \,\cos \,\theta )$ with the constant fitting parameter *C*_0_.

Combining the Eq. 2 with the expression for the jet power from^20^ and considering the jet diameter ${D}_{jet}$ being 1/10 of the capillary diameter *D*, we obtain the general expression:

3 $$\begin{array}{rcl}{P}_{jet} & \cong  & \frac{1}{8}\rho \pi {(D/10)}^{2}{\left({C}_{0}\frac{(E-{E}_{th})(1+\beta \cos \theta )}{ZD}\right)}^{3}\\  & \cong  & \frac{1}{8\cdot {10}^{2}}\rho \pi \frac{{{C}_{0}}^{3}}{{Z}^{3}}{(1+\beta \cos \theta )}^{3}\frac{1}{D}{(E-{E}_{th})}^{3}\end{array}$$

Therefore, the jet power is inversely proportional to the capillary diameter.

$${P}_{jet}\cong f(1/D)$$
